# Evaluation of the Performance and Ductility Index of Concrete Structures Using Advanced Composite Material Strengthening Methods

**DOI:** 10.3390/polym13234239

**Published:** 2021-12-03

**Authors:** Tae-Kyun Kim, Jong-Sup Park

**Affiliations:** Department of Structural Engineering Research, Korea Institute of Civil Engineering and Building Technology, 283, Goyang-daero, Ilsanseo-gu, Goyang-si 10223, Gyeonggi-do, Korea; jSpark1@kict.re.kr

**Keywords:** fiber reinforced polymer, strengthening methods, ductility index, brittle behavior, ductile behavior

## Abstract

The performance of concrete structures deteriorates over time. Thus, improving their performance using fiber-reinforced polymers (FRPs), PS strands, and various strengthening methods is important. Reinforced concrete (RC) and prestressed concrete (PSC) structures develop initial cracks in concrete during bending tests, and destruction occurs over a certain period of time after a certain load is generated, and then after the reinforcements and strands yield. However, in the case of FRP structures, after an initial concrete crack occurs, FRPs exhibit a rapid shape deformation of the structure after yielding. Thus, in this study we used FRP and PS strand materials and evaluated the ductility index using the load-displacement results obtained from structural tests conducted using various strengthening methods. The ductility index evaluation method compares and analyzes the change rates in the ductility index of PSC and RC structures based on a method that uses structural deflection and the derivation of the energy area ratio. The ductility evaluation results based on the energy area ratio at the crack, yield, and ultimate points showed that all the RC structures, except for the specimens strengthened with reinforcing materials from company H, were in the ductility and semi-ductility sections. Thus, all the PSC structures, except for the control specimens and PH4NP, were found to be brittle.

## 1. Introduction

Most social infrastructures and residential environments worldwide are composed of reinforced concrete (RC) and prestressed concrete (PSC) [1,2], which consist of materials such as concrete, steel reinforcement, and PS strands [1,2]. However, performance degradation occurs over time owing to the actions of the environment and external forces, and thus, structural reinforcement is required [3,4]. Various methods are available to improve the performance of structures. For example, the fiber reinforcement method primarily strengthens the lower parts of bridges and the interiors of tunnels by using glass, aramid and carbon fibers [5]. For fiber reinforcement, enabling integrated behavior between the sheet attached and the structure is the most important requirement; additionally, the bonding performance of the adhesive must be excellent [6,7]. Further, the attached fiber sheet can improve the strength of the member by sharing the tensile force inside the concrete via steel reinforcement and can also improve the flexural stiffness of the member and reduce the deformation of the steel reinforcement [6,7]. External prestressing (EP) is a construction method primarily applied to restore the load-carrying capacity of old bridges or to improve the load-carrying capacity of existing bridges owing to various changes in the use environment. The EP method can be applied to PSC box girder bridges, I-beam bridges, and steel plate girder bridges [8,9]. For the EP method, prestressing is introduced by installing anchorage devices for tendon installation and PS tendons on the outside of an existing concrete structure. In addition, the stress state of an existing member can be improved through EP. The benefits of EP are deflection prevention and crack control, and re-tensioning is simple [10,11]. However, in certain structures, the installation of anchorage devices for tendon installation can be subjected to spatial and environmental restrictions, and corrosion may occur over time because of environmental factors, such as salt damage and carbon dioxide, due to steel wires being exposed to the outside, which may further damage the appearance of the structure [12]. For the near-surface-mounted (NSM) method, reinforcement was initially performed using conventional steel reinforcement or plates; however, studies have been actively conducted using fiber-reinforced polymers (FRPs) [13]. In the case of the external bonding (EB) method, which is the most extensively used method, bond failure mostly occurs at the interface between the reinforcing material and concrete, which causes brittle failure behavior, and in certain cases, the performance of the reinforcing material is not fully exhibited [14,15]. In addition, the direct and indirect exposure of reinforcing materials to the outside may cause extreme loads of collision, explosion, fire, and easy exposure to various environmental conditions, such as salt damage, moisture, carbon dioxide, and damage [16]. In contrast, the NSM method can overcome the shortcomings of the conventional EB method employing the embedding and filling methods of reinforcing materials; however, it is necessary to enable the integrated behavior of epoxy and grout with the structure.

As for the materials used in the strengthening methods, FRPs are used to replace the steel reinforcement and PS strands, owing to their considerable benefits, including resistance to corrosion that may occur due to salt damage, carbonation and resistance to fire. However, they exhibit brittle behavior owing to the inherent properties of the materials [5], which can be quite dangerous. This is because the sudden brittle failure of structures may result in human casualties and property damage. Therefore, when reinforcement is performed using FRPs, it is necessary to maximize ductility while securing better performance than steel reinforcement and PS strands. Fantilli et al. evaluated the fiber content and ductility index of concrete beams. Abbass et al. experimentally evaluated the effect of steel fibers on the behavior and ductility of high-strength concrete hollow beams. Yang et al. experimentally studied the flexural behavior of high-strength fiber-reinforced concrete and high-strength concrete beams. Lee et al. evaluated the efficiency of supplementary V-ties for the flexural ductility of RC columns. Yang et al. evaluated the ductility of lightweight aggregate concrete T-beams reinforced with a maximum longitudinal reinforcement ratio [17,18,19,20,21]. This discussion shows that studies on compression members with FRP strengthening are numerous [22,23,24,25]. Further, various studies have been conducted on fiber incorporation and cement structures [26,27,28]. However, all existing studies have focused on evaluating the ductility index of structures that are primarily reinforced with steel and fibers rather than FRPs. Although studies on ductility are available, they remain insufficient.

Therefore, in this study we used FRP and PS strand materials and evaluated the ductility index using the load–displacement results based on the structural tests conducted using various strengthening methods [22,23]. Unlike previous studies that mainly evaluated the ductility index for RC structures, in this study, we compared and analyzed the RC and PSC structures and FRP-reinforced structures. In order to apply it to a real structure, we conducted a comparative analysis because it is necessary to first understand how FRP affects the structure, especially as a brittle material. The ductility index evaluation method compares and analyzes the change rates in the ductility index of PSC and RC structures based on a method that uses structural deflection and the derivation of the energy area ratio [22,23]. Moreover, based on this analysis, it is possible to set the optimal strengthening method according to the structure type and reinforcing material.

## 2. Materials and Methods

### 2.1. Advanced Composite Materials

FRPs have been used in several studies and fields. Most composite materials exhibit low density, high specific strength and specific stiffness, low thermal expansion coefficient, brittle failure, anisotropy with excellent strength and stiffness in the fiber direction, and high resistance to corrosion and fatigue [5]. In particular, the carbon fiber-reinforced polymers (CFRPs) used in this study exhibit high elasticity and strength due to the addition of carbon fibers to plastic materials, such as carbon fiber composite materials produced using carbon fibers as reinforcement and combining a matrix resin with plastic materials [5]. This is because, although carbon fibers have significantly lower density, higher tensile strength, higher modulus of elasticity, lower weight (a fourth of the weight of iron), and a lower thermal expansion rate than conventional iron, they are approximately 10 times stronger than iron. Thus, they have attracted attention as lightweight materials for application in automobile and aerospace industries, architecture, and various sporting goods. However, they are expensive [5].

### 2.2. Ductility Index Theory

**Figure 1:**
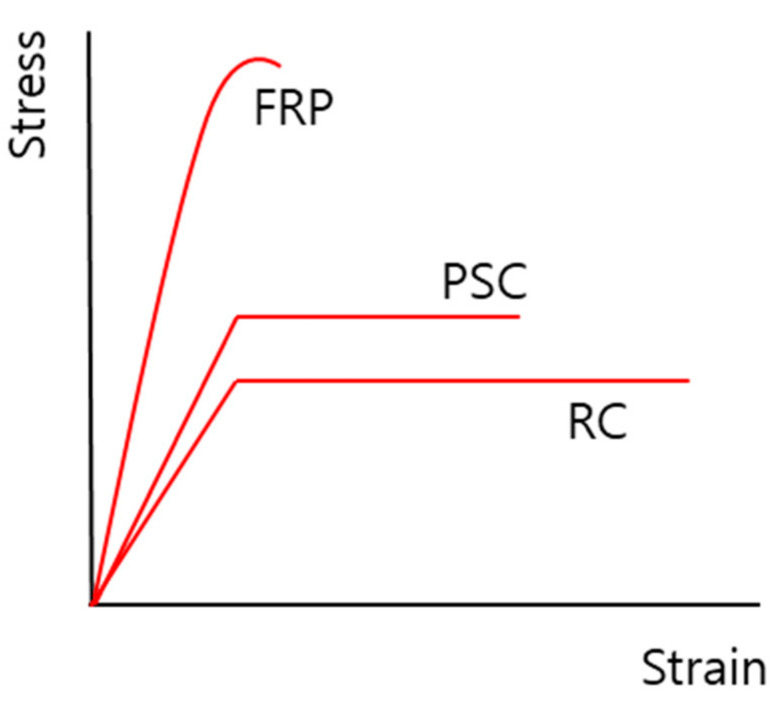
Stress–strain curve.

FRPs offer significant benefits when employed as a substitute for steel reinforcements; however, they exhibit brittle behavior. Therefore, when existing PSC and RC structures are reinforced with FRPs, maintaining the ductility index is important. Ductile behavior represents the range of the nonlinear section before failure when the material, member, and cross section of a structure are subjected to loading, as shown in Figure 1. PSC and RC structures exhibit a considerably wide range, but FRP structures fail simultaneously with yielding [29]. In general, RC and PSC structures develop initial cracks in concrete during bending tests, and destruction occurs over a certain period of time after a certain load is generated and then after the yield of reinforcement and strand. However, in the case of FRP structures, after the initial concrete crack occurs, FRP experiences a rapid shape deformation of the structure [29].

To define the general method to evaluate the ductility index, the ratios of the curvature $(\mu_{\Delta }$), rotation $\left(\mu_{\theta }\right)$, and deflection ($\mu_{\emptyset })$ can be expressed as deformation, as shown in Equation (1) [30]. In this study, the ductility index was evaluated using the ductility ratio via deflection based on the load-displacement test results:

(1) $$\mu_{\Delta }=\frac{\Delta_{u}}{\Delta_{y}},\;\mu_{\theta }=\frac{\theta_{u}}{\theta_{y}},\;\mu_{\emptyset }=\frac{\emptyset_{u}}{\emptyset_{y}}$$

**Figure 2:**
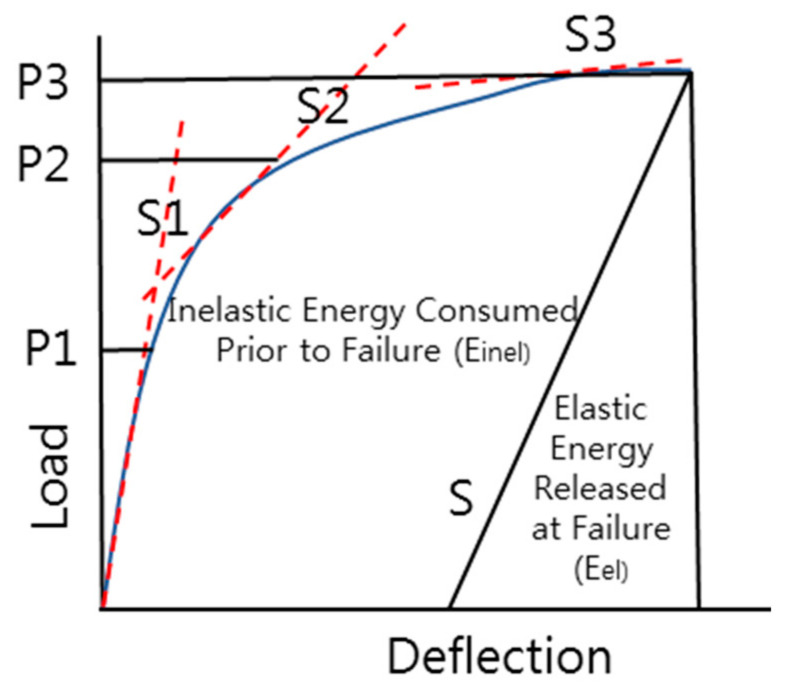
Energy area method theory.

In the case of RC, PSC, and steel structures, the definition of the yield state is clear because of the characteristics of steel [29]. Because the yield point is not clear for structures that use FRP members [30], the ductility index was derived using Equations (2)–(4) based on the energy area ratio theory proposed by Jeong in 1994, as shown in Figure 2 [30].

(2) $$E_{tot}=E_{inel}+E_{el}\;$$

(3) $$S=\frac{\left(P1S1+\left(P2-P1\right)S2+\left(P3-P2\right)S3\right)}{P3}$$

(4) $$\mu =\frac{\left(\frac{E_{tot}}{E_{el}}+1\right)}{2}$$

## 3. Ductility Index Evaluation

### 3.1. Prestressed Concrete (PSC) Structure

Table 1 shows the load-displacement test results of PSC structures reinforced with FRPs by Kim et al. [31,32,33]. Four-node tests were also conducted. Specimens 1–4 were control specimens, 5–6 were prestressed NSM specimens, 7–8 were non-prestressed NSM specimens, and 9–12 were EP specimens. Further, concrete strengths of 20 and 40 MPa were used. In addition, the displacement was measured at the crack, yield, and ultimate points. The stiffness up to the yield load increased in the cases of the PH4EP and PL4EP specimens by nearly twice that of the control specimen, but in the case of the NSM (no prestressing) specimen, there was no increase in stiffness and similar behavior can be observed in the case of the control specimen. From these results, the overall strengthening effect obtained was in the order of EP > NSM (prestressing) > NSM (no prestressing). Furthermore, the higher the stiffness was, the more brittle the structure was. However, in actual applications of concrete strengthening according to the proposed methods in this study, a gap was generated between the anchorage and the grooving when the NSP method was applied. With the application of the EP method, cracks occurred at the anchorage. Therefore, it is thought that EP and NSM methods will show improved strengthening effects when these practical problems are resolved [31].

Further, the ductility indices were derived at the yield and ultimate points using the load-displacement curve and the deflection ratio based on Equation (1). The ductility indices of control specimens PH4C and PL4C were 2.82 and 3.39, respectively, with a concrete compressive strength of 40 MPa, indicating that the ductility index of PL4C with low prestressing was approximately 1.2 times higher. In contrast, the ductility indices of PH2C and PL2C were 1.89 and 2.38, respectively, with a concrete compressive strength of 20 MPa, indicating that the ductility index of PL2C with low prestressing was approximately 1.25 times higher. Thus, for the PSC control specimens, the ductility index was found to increase as the concrete strength increased and prestressing decreased.

The ductility indices of NSM specimens PH4NP and PL4NP were 2.76 and 2.35, respectively, with a concrete compressive strength of 40 MPa, showing that the ductility index of PH4NP with low prestressing was approximately 1.17 times higher. The ductility indices of PL2NN(H) and PL2NN(S) were 2.56 and 2.27, respectively, with a concrete compressive strength of 20 MPa, showing that the ductility index of PL2NN(H), a specimen strengthened with the reinforcing material from manufacturer H, was approximately 1.12 times higher. For the NSM specimens, the ductility index was found to be slightly higher as the prestressing increased at a high concrete strength of 40 MPa, as opposed to the control specimens. Moreover, most of the NSM specimens exhibited similar ductility index ranges.

The ductility indices of EP specimens PH4EP and PL4EP were 1.70 and 1.50, respectively, with a concrete compressive strength of 40 MPa, showing that the ductility index of PH4EP with high prestressing was approximately 1.13 times higher. The ductility indices of PH2EP and PL2EP were 1.55 and 1.54, respectively, with a concrete compressive strength of 20 MPa, indicating that the ductility indices were almost identical regardless of prestressing. For the EP method, the reinforcement effect had no significant impact on the ductility index at low strength, but the ductility index increased as the prestressing increased at high strength.

Table 2 shows the ductility evaluation conducted by Grace in 1998 based on the energy concept using Equations (2)–(4) [25]. In terms of ductility energy area ratio, 0–69% represents brittleness, 70–74% represents semi-ductility, and 75–100% represents ductility. The ductility indices of control specimens PH4C, PL4C, PH2C, and PL2C were found to be 3.05, 3.01, 2.19, and 1.89, respectively. This indicated that the ductility index was approximately 1.5 times higher as the concrete strength increased, and it was slightly higher as the prestressing increased. Further, the energy ratio was found to be approximately 80% (ductility) for high-strength concrete specimens PH4C and PL4C, 70.38% (semi-ductility) for PH2C, and 64.06% (brittleness) for PL2C. The energy ratio results were similar to the ductility index results.

The ductility indices of the NSM specimens PH4NP, PL4NP, PL2NN(H), and PL2NN(S) were found to be 2.70, 1.96, 1.69, and 1.70, respectively. Regarding the control specimens, the ductility index was found to be 1.2 to 1.5 times higher as the concrete strength increased, and it was 1.35 times higher as prestressing increased. In addition, there was no significant change in the ductility index due to the reinforcing materials of companies H and S. Only PH4NP exhibited ductility at an energy area ratio of 77.24%, and the remaining PL4NP and PL2NN specimens exhibited brittleness when the ratio was between 55% and 65%. Moreover, the energy area ratio results were also found to be similar to the ductility index results. The ductility indices of EP specimens PH4EP, PL4EP, PH2EP, and PL2EP were found to be 2.07, 1.92, 1.59, and 1.52, respectively. Thus, the ductility index was also found to be 1.2 to 1.3 times higher as the concrete strength increased; however, the prestressing of the reinforcing material had no significant influence on the ductility index. Further, the energy area ratio was found to be approximately 10–15% higher for high-strength specimens PH4EP and PL4EP, but was generally between 50 and 69%, representing brittleness. The analysis of ductility indices in Table 2 and Table 3 revealed that the ductility evaluation using the energy area ratio at the crack, yield, and ultimate points could derive slightly more uniform results than the ductility index that used only the simple deflection at the yield and ultimate points. In addition, when additional prestressing was applied using FRPs and PS strands, the ductility index was reduced by a factor of approximately 1.2 to 2. However, it was confirmed that FRPs exhibited relatively higher ductility indices than the PS strands.

### 3.2. Comparative Analysis of Ductility Index Change Rates of PSC and RC Structures

Table 4 shows the results of ductility evaluation of PSC structures performed in this study and those of RC structures in a previous study [26]. As mentioned previously, previous studies mainly evaluated only the ductility index of RC structures; in this study, we compared and analyzed RC and PSC structures and FRP-reinforced structures. To apply it to a real structure, we conducted a comparative analysis because it is necessary to first understand how FRPs affect the structure, especially as a brittle material. Specimens 1 and 2 were control specimens, and 3 and 4 were NSM(P) specimens. Further, specimens 5 and 6 were NSM(N) specimens, and 7 and 8 were EP specimens. Table 3 compares the ductility index based on the displacement at the yield and ultimate points. In the case of the control specimens, the ductility index of the high-strength specimen R4C was found to be approximately 1.6 and 1.3 times higher than those of PH4C and PL4C, respectively. In contrast, the ductility index of the low-strength specimen R2C was approximately 3.9 and 3.1 times higher than those of PH2C and PL2C, respectively. Further, in the case of the NSM method, the ductility index of R4NSP with prestressing reinforcement was found to be approximately 2 and 2.4 times higher than those of PH4NP and PL4NP, respectively. For the specimens strengthened with reinforcing materials from companies H and S without the introduction of prestressing reinforcement, the ductility index of R2NSN(H) was approximately 0.79 times lower than that of PL2NN(H), and the ductility index of R2NSN(S) was 1.75 times higher than that of PL2NN(H). In addition, in the case of the H-reinforcing material, the RC specimen exhibited a lower ductility index. Further, in the case of the EP method, the ductility index of high-strength specimen R4EPP was almost identical to those of PH4EP and PL4EP, with a difference of 0.1. In addition, the ductility index of low-strength specimen R2EPP was 1.15 and 1.16 times higher than those of PH2EP and PL2EP, respectively. Thus, the ductility evaluation results based on the displacement at the yield and ultimate points showed that the ductility index of RC structures was higher than that of PSC structures for the control specimens. Furthermore, the ductility index of RC structures was higher for the NSM method, and both the RC and PSC specimens exhibited similar ductility indices for the EP method.

Table 4 presents the comparison of the ductility indices of the RC and PSC structures based on the energy area ratio at the crack, yield, and ultimate points. In the case of the control specimens, the ductility index of high-strength specimen R4C was found to be approximately 2.68 and 2.71 times higher than those of PH4C and PL4C, respectively. Further, the ductility index of low-strength specimen R2C was approximately 3.74 and 4.34 times higher than those of PH2C and PL2C, respectively. In the case of the NSM method, the ductility index of R4NSP with prestressing reinforcement introduced was found to be approximately 2.68 and 2.71 times higher than those of PH4NP and PL4NP, respectively. As for the specimens strengthened with reinforcing materials from companies H and S without the introduction of prestressing reinforcement, the ductility index of R2NSN(H) was approximately 1.08 times higher than that of PL2NN(H), while that of R2NSN(S) was 1.39 times higher than that of PL2NN(H). In the case of the EP method, the ductility index of high-strength specimen R4EPP was found to be 1.2 and 1.3 times higher than those of PH4EP and PL4EP, respectively. In addition, the ductility index of low-strength specimen R2EPP was 1.45 and 1.52 times higher than those of PH2EP and PL2EP, respectively.

The ductility evaluation results based on the energy area ratio showed that the ductility index of high-strength specimen R4C was approximately 1.16 and 1.17 times higher than those of PH4C and PL4C, respectively, for the control specimens, and that the energy area ratio ranged from 80 to 94% (ductility) for the specimens. Further, the ductility index of low-strength specimen R2C was approximately 1.33 and 1.46 times higher than those of PH2C and PL2C, respectively. Consequently, R2C was found to be in the ductility section, PH2C in the semi-ductility section, and PL2C in the brittleness section. In the case of the NSM method, the ductility index of R4NSP with prestressing reinforcement introduced was approximately 1.16 and 1.36 times higher than those of PH4NP and PL4NP, respectively. Thus, R4NSP and PH4NP were in the ductility section, and PL4NP was in the brittleness section. As for the specimens strengthened with reinforcing materials from companies H and S without the introduction of prestressing reinforcement, the ductility index of R2NSN(H) was approximately 1.07 times higher than that of PL2NN(H), and the ductility index of R2NSN(S) was 1.25 times higher than that of PL2NN(H). Consequently, R2NSN(H), PL2NN(H), R2NSN(S), and PL2NN(H) were found to be brittle, semi-ductile, brittle, and brittle, respectively. The ductility index of high-strength specimen R4EPP was 1.1 and 1.16 times higher than those of PH4EP and PL4EP, respectively. R4EPP, PH4EP, and PL4EP exhibited semi-ductility, and brittleness, respectively. The ductility index of low-strength R2EPP was 1.34 and 1.43 times higher than those of PH2EP and PL2EP, respectively. Thus, R2EPP, PH2EP, and PL2EP exhibited semi-ductility, brittleness, and brittleness, respectively.

The ductility evaluation results based on the energy area ratio at the crack, yield, and ultimate points showed that all of the RC structures, except for the specimens strengthened with reinforcing materials from company H, were in the ductility and semi-ductility sections. Thus, all the PSC structures, except for the control specimens and PH4NP, were found to be brittle.

## 4. Conclusions

In this study, various structural strengthening methods were applied using fiber-reinforced polymers (FRPs) and strands. Reinforced concrete (RC) and prestressed concrete (PSC) structures were compared based on the ductility index that used deflection at the yield and ultimate points and the ductility evaluation that used the energy area method at the crack, yield, and ultimate points. The following conclusions can be drawn.

When the PSC and RC structures were compared, the ductility index of PSC structures for the deflection ratio was approximately more than twice that of RC structures for the control specimens. The ductility index of RC structures was approximately twice as high as that of the NSM method (P), and the rest exhibited similar ductility indices. Regarding the ductility index for the energy area ratio concept, the RC structures exhibited higher values for the control specimens and all construction methods. Further, it was confirmed that most of the RC structures belonged to the ductility range, and most of the PSC structures were in the brittleness range. In this study, the ductility index was compared and analyzed using FRPs in relation to the NSM and EP methods of PSC and RC structures. In the future, it is expected that the optimal strengthening method can be set according to the structure type and reinforcing material by securing the ductile behavior of structures. However, to derive clearer and reliable data, further research is required on more diverse FRPs, such as glass fiber reinforced polymers (GFRPs) and aramid fiber-reinforced polymers (AFRPs), more strengthening methods, and various structural shape variables.

## Figures and Tables

**Table 1:** Load-displacement and ductility of PSC structures.

No.	Specimens	$\mathrm{Crack}\;\mathrm{Point}\;(P_{cr})$		$\mathrm{Yield}\;\mathrm{Point}\;(P_{y})$		$\mathrm{Ultimate}\;\mathrm{Point}\;(P_{u})$		Ductility Index (D)
		Load (kN)	Disp. (mm)	Load (kN)	Disp. (mm)	Load (kN)	Disp. (mm)	
1	PH4C	94.5	5.90	218.8	28.14	237.6	79.35	2.82
2	PL4C	63.7	3.14	185.5	28.98	239.3	98.10	3.39
3	PH2C	83.3	5.58	219.6	33.29	233.3	63.03	1.89
4	PL2C	50.6	3.57	180.4	30.17	227.3	71.76	2.38
5	PH4NP	125.6	6.60	273.3	28.89	312.8	79.80	2.76
6	PL4NP	90.2	4.29	232.0	27.01	300.0	63.48	2.35
7	PL2NN(H)	46.2	3.36	198.1	32.32	271.4	82.64	2.56
8	PL2NN(S)	42.6	2.64	188.8	31.75	248.7	72.12	2.27
9	PH4EP	141.7	5.62	323.9	33.30	356.4	56.49	1.70
10	PL4EP	132.1	4.95	311.3	31.72	336.8	47.43	1.50
11	PH2EP	98.6	6.04	285.9	37.16	317.0	57.66	1.55
12	PL2EP	108.4	5.81	275.8	36.91	335.3	56.70	1.54

**Table 2:** Ductility of energy ratio method for PSC structures.

No.	Specimens	Energy				Analysis	Ductility Index(E)
		Total	Elastic	Inelastic	Rate		
1	PH4C	15,448.79	3027.56	12,421.23	80.40	D	3.05
2	PL4C	18,000.76	3590.56	14,410.20	80.05	D	3.01
3	PH2C	11,163.71	3306.37	7857.34	70.38	SD	2.19
4	PL2C	11,640.74	4183.77	7456.97	64.06	B	1.89
5	PH4NP	19,779.40	4501.40	15,277.99	77.24	D	2.70
6	PL4NP	13,554.69	4641.88	8912.81	65.75	B	1.96
7	PL2NN(H)	15,427.70	6495.72	8931.98	57.90	B	1.69
8	PL2NN(S)	12,255.20	5091.69	7163.51	58.45	B	1.70
9	PH4EP	14,730.16	4698.41	10,031.75	68.10	B	2.07
10	PL4EP	11,352.68	4007.79	7344.89	64.70	B	1.92
11	PH2EP	12,460.32	5720.95	6739.37	54.09	B	1.59
12	PL2EP	12,336.05	6075.34	6260.71	50.75	B	1.52

D = ductile, SD = semi-ductile, B = brittle.

**Table 3:** Ductility evaluation comparison of deflection for RC and PSC structures.

No.	RC Specimens	RC Ductility Index	PSC Specimens	PSC Ductility	PSC Specimens	PSC Ductility
1	R4C	4.39	PH4C	2.82	PL4C	3.39
2	R2C	7.32	PH2C	1.89	PL2C	2.38
3	R4NSP	5.58	PH4NP	2.76	PL4NP	2.35
4	R2NSP	3.87	-		-	
5	R2NSN(H)	2.02	-		PL2NN(H)	2.56
6	R2NSN(S)	3.98	-		PL2NN(S)	2.27
7	R4EPP	1.60	PH4EP	1.70	PL4EP	1.50
8	R2EPP	1.79	PH2EP	1.55	PL2EP	1.54
9	R4EBN	1.82	-		-	
10	R2EBN	1.92	-		-	

**Table 4:** Comparison of ductility indices of RC and PSC structures based on energy ratio method.

No.	RC			PSC					
	Specimens	Energy Rate	Analysis	Specimens	Energy Rate	Analysis	Specimens	Energy Rate	Analysis
1	R4C	93.47(D)	8.16	PH4C	80.40(D)	3.05	PL4C	80.05(D)	3.01
2	R2C	93.51(D)	8.20	PH2C	70.38(SD)	2.19	PL2C	64.06(B)	1.89
3	R4NSP	89.60(D)	5.31	PH4NP	77.24(D)	2.70	PL4NP	65.75(B)	1.96
4	R2NSP	84.69(D)	3.77	-	-	-	-	-	-
5	R2NSN(H)	62.11(B)	1.82				PL2NN(H)	57.90(B)	1.69
6	R4NSN(S)	73.12(SD)	2.36				PL2NN(S)	58.45(B)	1.70
7	R4EPP	74.86(SD)	2.49	PH4EP	68.10(B)	2.07	PL4EP	64.70(B)	1.92
8	R2EPP	72.40(SD)	2.31	PH2EP	54.09(B)	1.59	PL2EP	50.75(B)	1.52
9	R4EBN	76.38(D)	2.62	-	-	-	-	-	-
10	R2EBN	49.77(B)	1.50	-	-	-	-	-	-

D = ductile, SD = semi-ductile, B = brittle.

## Data Availability

The data presented in this study are available upon request from the corresponding author.
